# Lesion-aware attention network for diabetic nephropathy diagnosis with optical coherence tomography images

**DOI:** 10.3389/fmed.2023.1259478

**Published:** 2023-10-27

**Authors:** Yuliang Liu, Fenghang Zhang, Xizhan Gao, Tingting Liu, Jiwen Dong

**Affiliations:** ^1^School of Information Science and Engineering, University of Jinan, Jinan, China; ^2^Shandong Provincial Key Laboratory of Network-Based Intelligent Computing, Jinan, China; ^3^Shandong Eye Institute, Shandong First Medical University and Shandong Academy of Medical Sciences, Jinan, China

**Keywords:** nephropathy retinopathy, retinal image classification, convolutional neural networks, attention mechanisms, auxiliary diagnosis

## Abstract

**Purpose:**

For early screening of diabetic nephropathy patients, we propose a deep learning algorithm to screen high-risk patients with diabetic nephropathy from retinal images of diabetic patients.

**Methods:**

We propose the use of attentional mechanisms to improve the model’s focus on lesion-prone regions of retinal OCT images. First, the data is trained using the base network and the Grad-CAM algorithm locates image regions that have a large impact on the model output and generates a rough mask localization map. The mask is used as a auxiliary region to realize the auxiliary attention module. We then inserted the region-guided attention module into the baseline model and trained the CNN model to guide the model to better focus on relevant lesion features. The proposed model improves the recognition of the lesion region.

**Results:**

To evaluate the lesion-aware attention network, we trained and tested it using OCT volumetric data collected from 66 patients with diabetic retinal microangiopathy (89 eyes, male = 43, female = 23). There were 45 patients (60 eyes, male=27, female = 18) in DR group and 21 patients (29 eyes, male = 16, female = 5) in DN group. Our proposed model performs even better in disease classification, specifically, the accuracy of the proposed model was 91.68%, the sensitivity was 89.99%, and the specificity was 92.18%.

**Conclusion:**

The proposed lesion-aware attention model can provide reliable screening of high-risk patients with diabetic nephropathy.

## 1. Introduction

Diabetes mellitus, a prevalent chronic disease accompanied by metabolic disorders, is one of the greatest public health challenges of the 21st century (1, 2). Over the past few decades, there has been a persistent upward trend in its prevalence and incidence (3). According to the International Diabetes Federation (IDF) 2021, 530 million people worldwide have diabetes (4). Diabetes not only has an impact on the quality of life for patients, but if not properly treated and managed, it can also give rise to a range of severe microvascular complications (5, 6). Diabetic nephropathy (DN) and diabetic retinopathy (DR) are the common complication of diabetic mellitus (7).

DN is one of the leading cause of end-stage renal disease (ESRD) (8), and the mortality rate is about 30 times higher than that of diabetic patients without DN (9). Therefore, it is extremely important to screen diabetic patients at risk of DN for improving prognosis and slow down the progress of the disease. However, there are a large number of people with diabetes and medical resources are limited. This is undoubtedly a challenge for effective disease screening and management.

Diabetes can cause changes in the retina to some extent, and the degree of lesions in the retinal image correlates with the disease progression of diabetes (10). There were studies showing that diabetic patients with severe retinal lesions were at risk of developing DN (11). This offers the potential to achieve early screening of DN by analyzing retinal images. Moreover, retinal image screening is non-invasive and reduces the time and cost of screening compared to other tests such as blood tests or kidney biopsies (12).

In recent years, the combination of artificial intelligence models and medical images has been increasingly used in clinical practice. For example, magnetic resonance (MR) images are an important tool for clinical exploration of brain structure. You et al. (13) proposed fine perceptual generative adversarial networks (FP-GANs) to generate super-resolution (SR) MR images from a low-resolution counterpart network. Hu et al. (14) proposed a bidirectional mapping generative adversarial networks (BMGAN). This is a 3D end-to-end synthesis network that effectively utilizes image context and latent vectors for brain MR to PET synthesis. In (15), the authors proposed a novel network called consistent perception generative adversarial network (CPGAN) for a semi-supervised stroke injury segmentation task, which can reduce the reliance on fully labeled samples.

Ophthalmic imaging is the primary method of assisting in the diagnosis and treatment of ophthalmic diseases. Deep learning models can be used not only to detect ocular diseases (age-related macular degeneration, diabetic retinopathy, etc.) (16–18), but also to screen for systemic diseases (19). In recent work, Kang et al. (20) evaluated a deep learning model, VGG-19, for detecting early renal impairment using retinal fundus images and showed that the model was more accurate in patients with elevated serum HbA1c levels. Zhao et al. (21) used a deep learning system based on lesion perception to classify diabetic retinopathy and used the classification results to further predict whether the patient would develop advanced diabetic kidney disease. In (22), Sabanayagam et al. developed and validated a deep learning algorithm based on retinal images to detect chronic kidney disease from retinal photographs of a community population.

However, it may be difficult for conventional convolutional neural networks to capture some of the complex and variable clinical representations of retinopathy. For example, the macular region is the most sensitive area of vision and plays a pivotal role in human visual function (23). It also largely reflects the major manifestations of DN (24, 25). In this case, the lesioned area may occupy only a small part of the image, which can lead to a less accurate identification of the lesion. In recent years, deep learning models with attention mechanisms have emerged to enhance the accuracy and efficiency of lesion detection. These models are capable of precisely focusing on the region of interest, thus improving the overall performance of the detection process. The main idea of the attention mechanism in deep learning is to highlight the discriminative regions of the feature map while suppressing irrelevant information (26). In this study, we propose a lesion-aware attention mechanism that adaptively adjusts the attention weights based on the feature information of the lesion region in the image. This mechanism can identify the lesion features more accurately and improve the classification accuracy of the model.

In general, our contributions can be summarized as follows:

1. We use the Grad-CAM algorithm to perform preliminary localization of lesion regions in OCT images as region guidance information.
2. We propose a lesion-aware attention module to learn relevant features from OCT images and avoid the interference of irrelevant region information.
3. Retinal OCT images collected in the clinic were used to evaluate our model. The experimental results show that our model can be effectively used for screening of DN.

## 2. Materials and methods

### 2.1. Dataset

The validity of our proposed model was evaluated using OCT datasets obtained from clinical practice. The data for this study were collected using the AngioVue system (Optovue RTVue XR100 Avanti; Optovue, Inc., Fremont, CA, United Stares) at Shandong Eye Hospital. To ensure the quality and accuracy of the images, patients in our study were screened by renal laboratory tests or by ophthalmologists. A total of 35,600 OCT images were collected from a sample of 66 patients (89 eyes) with diabetes mellitus. The patients were divided into DR group and DN group.

Inclusion criteria of DR group: (1) over 18 years old; (2) the patient was diagnosed with type 2 diabetes mellitus (T2DM) (27), but there are no other systemic symptoms. (3) The changes of retinal microvascular system met the diagnostic criteria of DR (28).

Inclusion criteria of DN group: (1) over 18 years old; (2) the patient was diagnosed with T2DM (27); (3) the patient was diagnosed with DN (29); (4) no symptoms of systemic diseases except T2DM or DN; (5) the changes of retinal microvascular system met the diagnostic criteria of DR (28).

Exclusion criteria included: (1) poor quality of OCT imaging; (2) there were other eye diseases besides DR; (3) the general condition was poor; (4) pregnancy or breastfeeding.

**Figure 1:**
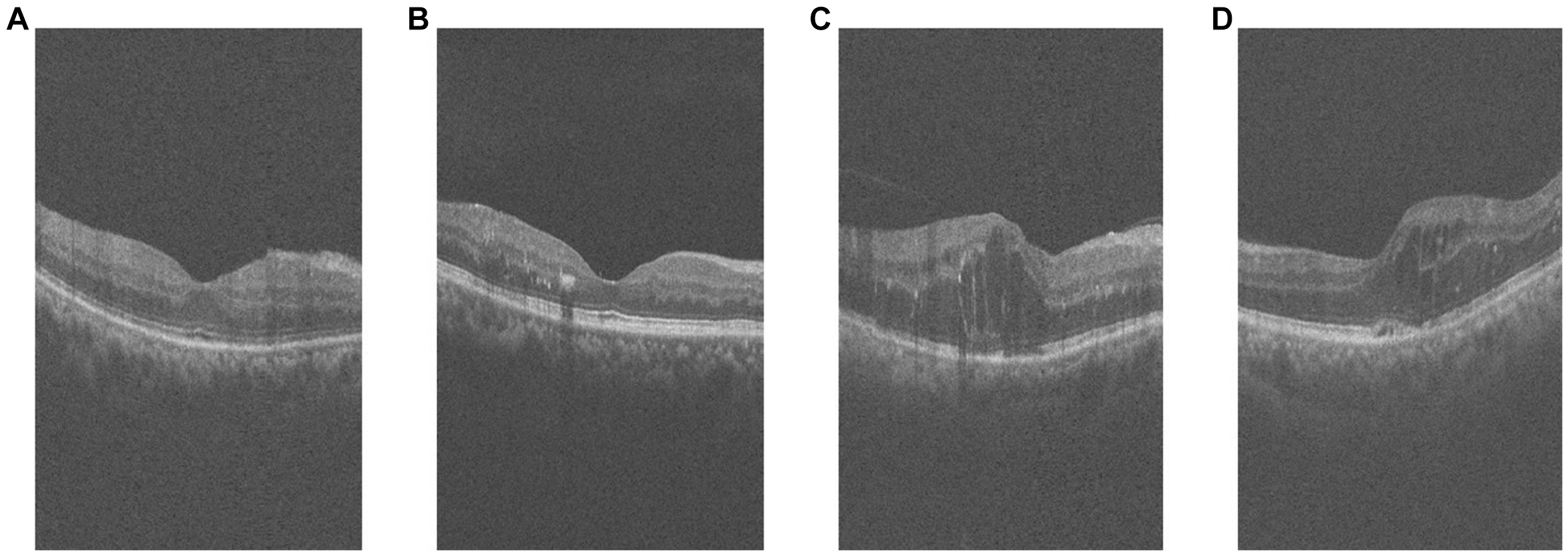
Example of retinal OCT images. **(A,B)** are OCT images of DR group, **(C,D)** are OCT images of DN group.

Among them, the comparison of OCT images of DR group and DN group is shown in Figure 1. The original size of each image was 400 × 640, and 400 2D cross-sectional images were taken for each eye of each patient, which could be used to visualize different parts and structures of the eye.

### 2.2. Data preprocessing

**Figure 2:**
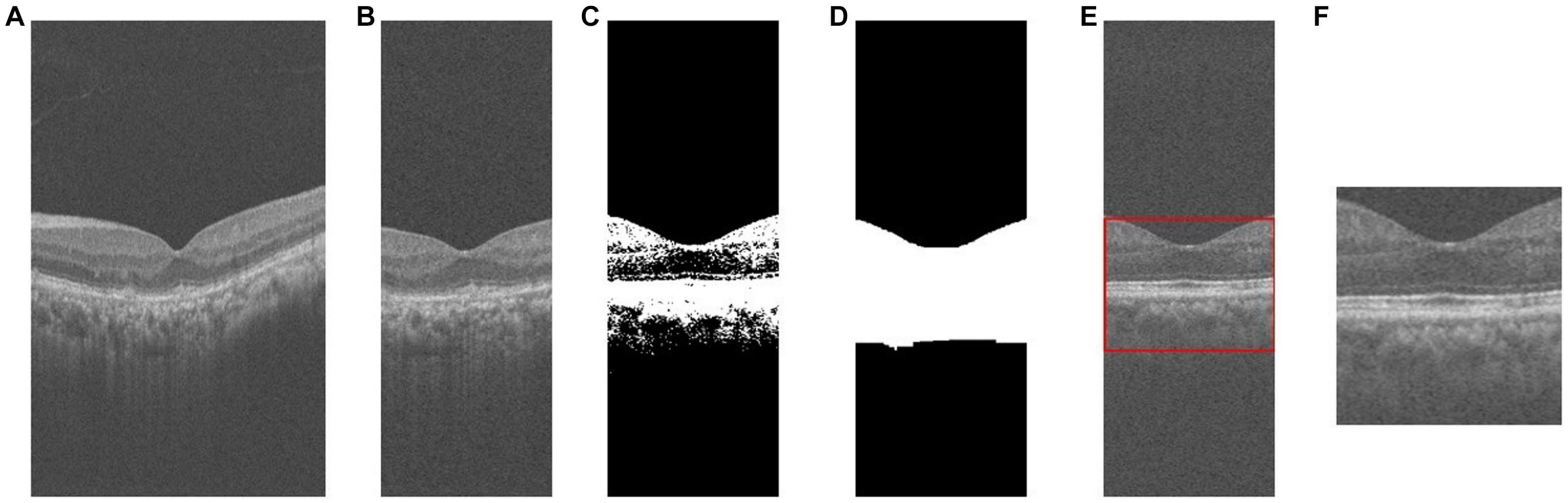
The process of OCT image pre-processing. **(A)** original image; **(B)** crop; **(C)** binarization; **(D)** morphological operations; **(E)** contour extraction; **(F)** foreground extraction.

During the image preprocessing, we considered cropping out the macular region of the image. First, we extracted a subset of images near the macular area of the patient’s retina by considering factors such as the length of the macular area and image spacing. Specifically, we selected the 130th to 270th out of 400 2D cross-sectional retinal images, resulting in a total of 140 images. And based on this, we sampled these 140 images at intervals to obtain 70 images for each patient to reduce the data volume and computational cost. Second, for each retinal OCT image, the central macular concavity is located in the central region of the retina, and we preserve the macular region by cropping it in the center of the image. Cropping its middle 140 pixels along the horizontal axis resized the 400 × 640 image to 140 × 640. Subsequently, in order to better extract feature information and minimize the impact of the black background, we employ a binarization process on the retinal image. Furthermore, to ensure the completeness and connectedness of the foreground in the retinal image, we also perform a morphological expansion operation. And the contour detection is performed on the morphologically inflated image to extract the foreground information of the retinal image. The specific preprocessing flow is shown in Figure 2. Finally, our screening is patient-based, and multiple OCT images of a patient are combined into one 3D image as a classification sample. That is, 70 2D OCT images of each patient are stacked into one 3D data to obtain a volumetric data of size 100 × 100 × 70.

## 3. Deep learning models

Our model is based on a 3D convolutional neural network (CNN). A 3D convolutional neural network is composed of convolutional layers, pooling layers and fully connected layers, etc., and is capable of feature extraction and classification of the input data. Compared with 2D CNNs, 3D CNNs can learn more complex feature information in 3D images. ResNet is a deep convolutional neural network, proposed by He et al. (30) in 2015. It is composed of residual blocks, and each residual block contains jump connections across layers so that the output of the residual block is the sum of the original input and the convolutional result. Such a design allows the network to propagate gradient information efficiently even at later stages, thus improving the training efficiency and performance of the network. Our study is based on the 3D ResNet architecture.

Existing retinal OCT image classification models usually learn the whole image, while in clinical diagnosis, ophthalmologists usually focus on the lesion area of the OCT image. The attention mechanism in deep learning allows the network to have different weights for the attention of different parts of the input data, thus improving the model’s recognition of critical information. Therefore, in order to simulate the clinical diagnosis of ophthalmologists, we propose using an attention mechanism to enhance the model’s focus on lesion-prone regions of retinal OCT images. A lesion aware attention mechanism is introduced, which enables the CNN model to better capture lesion features and learn lesion regions more accurately. This approach utilizing lesion regions as auxiliary information ultimately improves the classification performance of the model.

**Figure 3:**
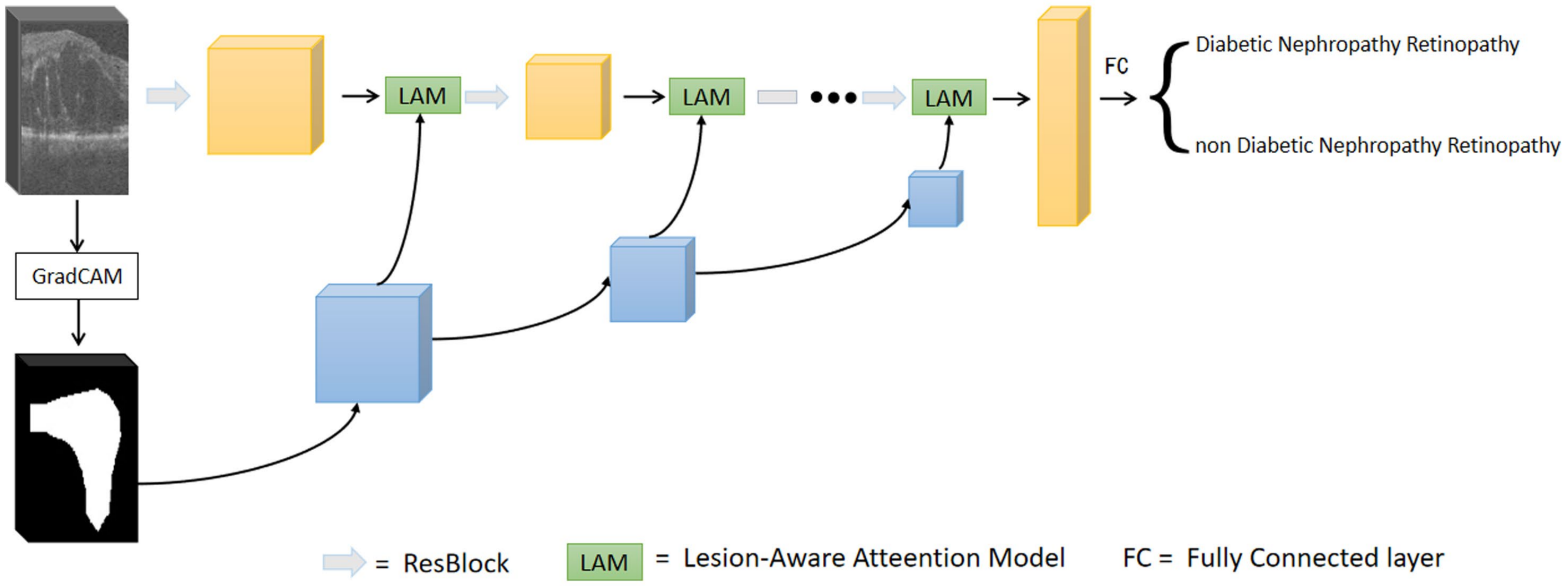
Overall network of lesion aware attention models.

The overall framework of the model is shown in Figure 3. The first step is to train the data using the base network, and to calculate the output gradient of the model through the Grad-CAM algorithm (31). This algorithm locates the image regions that have a large impact on the model output, which in turn generates a heat map. The heat map is binarized to generate a rough mask localization map to highlight the important regions in the image used for prediction, which is used as a guide region to implement the guided attention module. In the second step, we insert the region guided attention module into the baseline model, train the CNN model to guide the model to focus on and enhance the lesion regions. In the testing phase, the test data are fed into the trained model to get the classification results.

### 3.1. Generation of attentional regions

We used ResNet as the base network and input the preprocessed OCT images of DR group and DN group into the network for training. The trained model is used to generate the initial attention map. Based on the Grad-CAM algorithm (31), the class-discriminative localization map Grad-CAM $L_{\mathrm{Grad}-CAM}^{c}\in R^{u\times v}$ is obtained for any class c, where u is the width and v is the height. The gradient of the score $y_{c}$ is calculated with respect to the feature maps $A_{l,k}$ of the convolutional layer, i.e.,$\frac{\partial y_{c}}{\partial A_{l,k}}$. These gradients flowing back are global-average-pooled over the width and height dimensions (indexed by i and j respectively) to obtain the neuron importance weights $\alpha_{k}^{c}$ defined in Eq. 1 (31):


(1) $$\alpha_{k}^{c}=\frac{1}{Z}\underset{i}{\sum }\underset{j}{\sum }\frac{\partial y^{c}}{\partial A_{i,j}^{k}}$$

where Z is the product of the width and height of the feature layer.

The weight $\alpha_{k}^{c}$ serves to partially linearize the deep network from A, and captures the most discriminative part of the feature map for a given class c(31). We perform a weighted combination of forward activation maps followed by ReLU to obtain $L_{\mathrm{Grad}-CAM}^{c}$:


(2) $$L_{\mathrm{Grad}:CAM}^{c}=\mathrm{ReLU}\left(\sum \alpha_{k}^{c}A^{k}\right)$$

**Figure 4:**
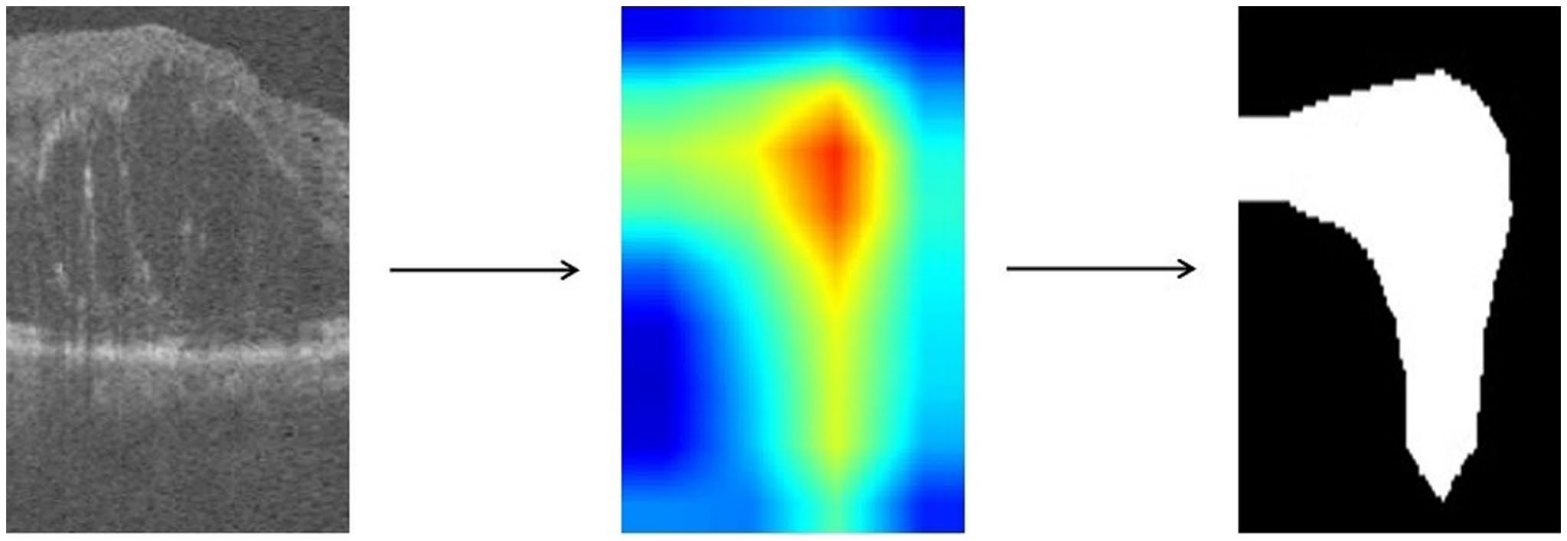
Heatmap of OCT image is obtained by Grad-CAM algorithm and processed to binary map.

For images in which retinopathy occurs, the algorithm can help capture the lesion features and render them as intense heat map regions. As shown in the Figure 4, the color distribution of the heat map typically indicates the importance of each pixel in the feature map for the model output. The red color indicates regions of higher importance and the blue color indicates regions of lower importance.

We take the grayscale map of the R channel in the heatmap and convert all pixel values in the grayscale map to 0 or 1 by thresholding to achieve the binarization process. Defined as in Eq. 3, with the threshold T set to 50.


(3) $$B\left(x,y\right)=\{\begin{array}{l} 0,\;\mathrm{if}\;I\left(x,y\right)<T\\ 1,\;\mathrm{otherwise} \end{array}$$

$I\left(x,y\right)$ represents the pixel value of the input grayscale map at position $\left(x,y\right)$, and $B\left(x,y\right)$ represents the pixel value of the output binary image at position $\left(x,y\right)$. T is the selected threshold value, and $B\left(x,y\right)$ takes the value of 1 when $I\left(x,y\right)$ is greater than T; otherwise, $B\left(x,y\right)$ takes the value of 0. The resulting binary map can highlight the target regions and visually demonstrate which areas in the image the model has focused on, facilitating further research and analysis.

### 3.2. Lesion-aware attention model

**Figure 5:**
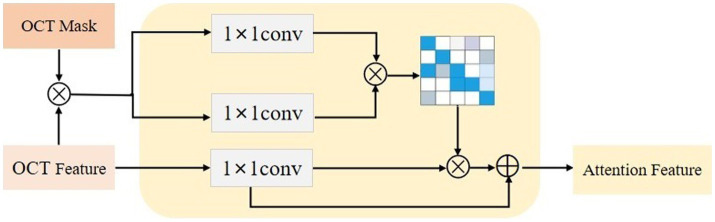
The lesion aware attention module.

After obtaining the region assisted information, the mask image is introduced into the attention mechanism and we propose a lesion area guided attention module to guide the model to better focus on the lesion region. The module is shown in Figure 5. Inspired by the self-attention mechanism (32), the region information is integrated into the self-attention module. Specifically, the feature map X of the OCT image and the mask image features $X_{B}$ are multiplied as input, and the lesion region is preserved to obtain the lesion-related feature $X_{L}$. We calculate the attentional weights using the lesion-related features $X_{L}$ and overlay the attentional weights to X to obtain the attentional features $A_{L}$ for lesion region perception as shown in Eq. 4. $X^{C}$ and $X_{L}^{C}$ represent the tensors that are generated by feeding X and $X_{L}$ into a 1×1 convolution operation, respectively.


(4) $$A_{L}=LAM\left(X,X_{L}\right)=\mathrm{softmax}\left(X_{L}^{C}X_{L}^{C^{T}}\right)X^{C}+XC$$

Since the mask is only a rough localization of the lesion region, the original features are added to avoid the loss of information. In our experiments, we insert the region-guided attention module after each residual block in ResNet18 to form the attention features to classify the OCT images.

### 3.3. Model training

We developed our model using PyTorch (33), and conducted experiments on NVIDIA Tesla T4. The OCT data was standardized to a size of 100 × 100 × 70 during model training. A data enhancement strategy of random rotation and random scaling was applied to the input images, and normalization was performed.

We trained the network using Adam optimizer (34) and optimized the model by means of a cross-entropy loss function. The initial learning rate was set to 10^−4^, and the learning rate was updated using the learning rate scheduler StepLR. Our network was trained with 150 epochs and the batch size was set to 2.

### 3.4. Statistical analysis

To evaluate the performance of the model in the classification task, we used three classification metrics, namely accuracy, sensitivity and specificity. The accuracy of the model is the number of correctly predicted samples as a percentage of the total number of samples. Sensitivity indicates the proportion of samples that are truly positive that are correctly identified, i.e., the probability of being judged as diseased among patients with the disease. Specificity indicates the proportion of samples that are truly negative that are correctly identified, i.e., the probability of being judged healthy among healthy individuals. They are defined as follows:


$$\mathrm{Accuracy}=\frac{TP+TN}{TP+FN+TN+FP}$$

$$\mathrm{Sensitivity}=\frac{TP}{TP+FN}$$

$$\mathrm{Specificity}=\frac{TN}{TN+FP}$$

TP denotes the number of samples with a true prediction of positive, FN denotes the number of samples with an error among those with a true prediction of negative, TN denotes the number of samples with a true prediction of negative, and FP denotes the number of samples with an error among those with a true prediction of positive.

## 4. Result

In our experiments, training and testing were performed using collected OCT volumetric data of 66 diabetic patients (89 eyes, male = 43, female = 23) with retinal microvascular lesions, which consisted of 45 patients (60 eyes, male = 27, female = 18) in DR group and 21 patients (29 eyes, male = 16, female = 5) in DN group. The basic clinical information of patients was shown in Table 1. In terms of gender, there was no statistical difference between the two groups. For age, the average ages of patients in DR group and DN group were 60.40 ± 8.63 (mean ± SD, age range: 42–80) and 56.65 ± 11.94 (mean ± SD, age range: 26–73), and there was no statistical difference between the two groups(*t* = 1.431, *p* = 0.157 > 0.05).

**Table 1:** Clinical characteristics of enrolled patients (mean ± SD).

Variable	DR group	DN group	(*P*/*χ*^2^ or *t* value)
*N* (subjects/eyes)	45/60	21/29	
Gender (male/female)	27/18	16/5	*χ*^2^ = 1.653
			*p* = 0.199
Age (years)	60.40 ± 8.63	56.65 ± 11.94	*t* = 1.431
			^*^p = 0.157
Duration of diabetes (years)	9.56 ± 4.30	14.19 ± 4.26	*t* = −4.104
			^***^p < 0.001
BCVA (LogMAR)	0.44 ± 0.29	0.73 ± 0.57	*t* = −2.568
			^*^p = 0.015

DR, diabetic retinopathy; DN, diabetic nephropathy; *N*, number; BCVA, best corrected visual acuity. ^*^p ≤ 0.05, there is a statistical difference; ^**^p ≤ 0.01, ^***^p ≤ 0.001, there is a significant difference.

The duration of diabetes in DR group (mean ± SD = 10.00 ± 5.77) was significantly shorter than that in DN group (mean ± SD = 14.05 ± 6.32) (*t* = −4.104, *p* < 0.001). The best corrected visual acuity (BCVA) was obtained by using the international standard visual acuity chart, and the results were recorded as the logarithmic minimum angle of resolution (LogMAR). The BCVA (LogMAR) of patients in DR group and DN group were 0.44 ± 0.29 and 0.73 ± 0.57 (mean ± SD). The BCVA (LogMAR) of DN group were worse than that of DR group (*t* = −2.568, *p* = 0.015 < 0.05).

**Figure 6:**
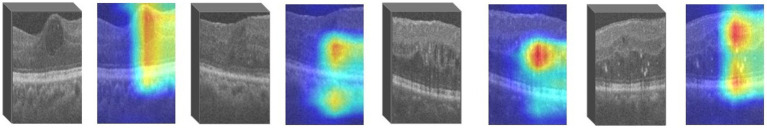
Rough localization of the lesion area using Grad-CAM.

We use ResNet18 as the baseline model, train the experimental data, and visualize the prediction results of the model using the Grad-CAM method. As shown in the Figure 6, the heatmap obtained by Grad-CAM was able to roughly localize the lesion region in the retinopathy images initially.

During the training of our model, due to the small amount of data in the dataset, the model will cause the overall accuracy to drop by about 4% to 5% if a sample is wrongly divided during the classification. Therefore, the performance of the model may be greatly affected in the case of a small test set. To improve the stability and accuracy of the model, we use a 5-fold cross-validation method to evaluate the performance of the model. The data set is randomly divided into five subsets, each subset is used as the test set in turn, and the remaining four subsets are used as the training set, with no overlap between the validation and training sets. That is, 80% of the data is used for training and the other 20% is used for testing, and the performance of the trained model is evaluated on the test set. The above steps are repeated five times to obtain five sets of training and testing results, and the average of these five sets of results is used as the evaluation index of the model performance.

**Figure 7:**
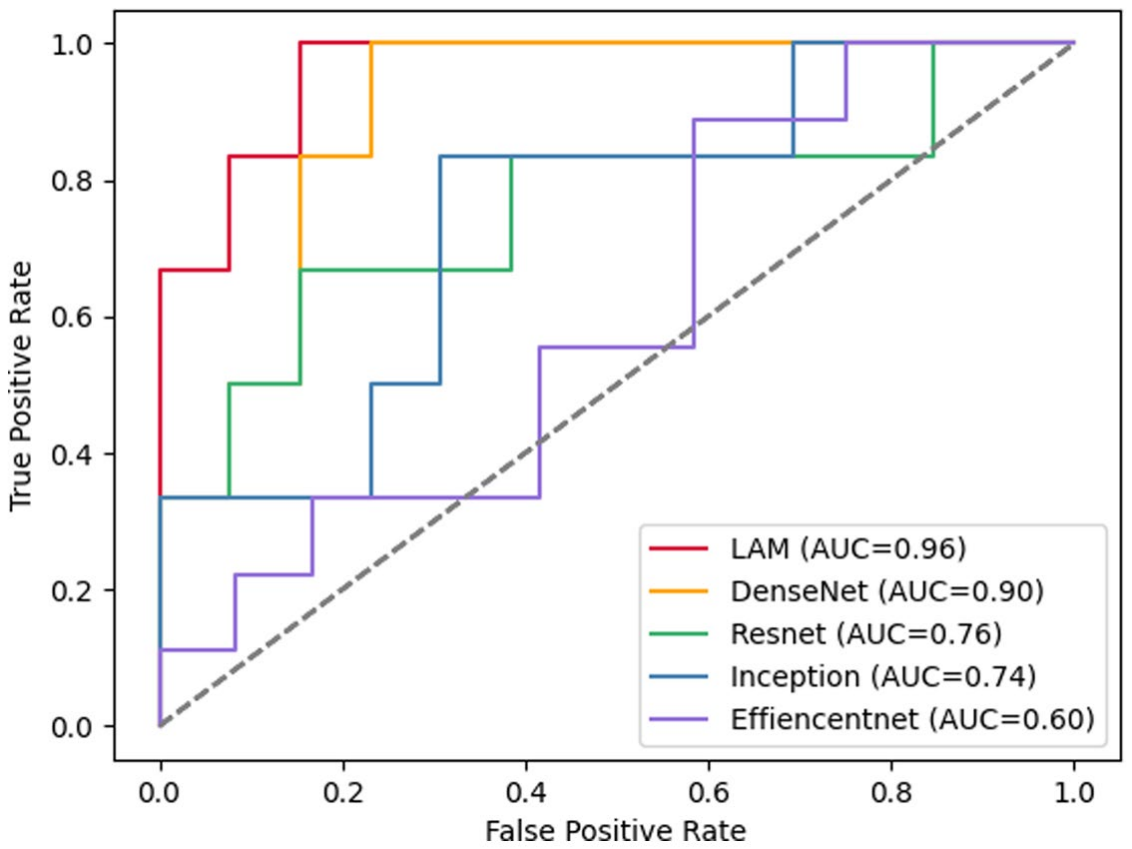
ROC plot of each deep learning model for retinal OCT image classification.

First, we compared it with the baseline model (ResNet18). The results are shown in Table 2. For the OCT images of DR group and DN group classification task, the baseline model has an accuracy of 84.51%, and sensitivity and specificity of 74.44% and 88.89%, respectively. Our proposed lesion aware attention model performs even better in disease classification, specifically, the accuracy of the proposed model was 91.68%, the sensitivity was 89.99%, and the specificity was 92.18%. In addition, we also used some recent classification models, DenseNet, InceptionV3 and EfficientNet to train and test on the dataset, and the models achieved accuracies of 82.30%, 83.35% and 78.39% on the test set, respectively. Among them, the InceptionV3 model performed the best, reaching the highest accuracy of 83.35%. However, the performance of EfficientNet is slightly inferior to the first two. This may be due to the fact that EfficientNet was designed to improve the computational efficiency and the number of parameters, while other models may have more advantages compared to it with full consideration of the model accuracy. Generate ROC curves using these five models, with the ROC plot shown in Figure 7.

**Table 2:** Comparison of our approach with other models. The best results in this table are marked in bold.

Model	Accuracy	Sensitivity	Specificity
ResNet	84.51	74.44	88.89
Densent	82.30	86.66	79.74
InceptionV3	83.35	81.11	84.49
Effiencentnet	78.39	54.44	86.64
The proposed model	**91.68**	**89.99**	**92.18**

These data further demonstrate the effectiveness and reliability of our method. Compared with previous classification models, our method is able to better focus on important regions and extract more discriminative features, thus improving the classification performance.

To further validate the effectiveness of our proposed method, we conducted ablation experiments for different models. Specifically, ResNet34, ResNet50, and ResNet101 were selected as the base models. Experiments are performed on data sets, using the same training and test Settings. Among them, the hyperparameters and data enhancement techniques used are also consistent with the previous experiments. The following experiments are conducted for different models: using the original ResNet34, ResNet50 and ResNet101 models as the baseline, and adding the lesion aware attention module after the residual blocks of ResNet34, ResNet50 and ResNet101.

The results of quantitative experiments are shown in Table 3:

**Table 3:** Quantitative experimental classification results. The best results in this table are marked in bold.

Model	Accuracy	Sensitivity	Specificity
ResNet18	84.51	74.44	88.89
ResNet18 + LAM	91.68	89.99	**92.18**
ResNet34	85.55	79.77	89.77
ResNet34 + LAM	**91.88**	**91.11**	92.05
ResNet50	82.50	78.88	84.35
ResNet50 + LAM	87.66	87.77	87.43
ResNet101	82.60	73.33	87.43
ResNet101 + LAM	85.55	84.44	85.89

We found that for the classification of retinal OCT images in the DR and DN groups, the ResNet model with the lesion-aware attention module was better. In terms of the baseline model, ResNet34 was the best, with a accuracy at 85.55%, followed by ResNet18 with a accuracy of 84.51%. However, the ResNet50 and ResNet101 models with more layers and more parameters do not bring advantages, with accuracy of around 82% for both, probably because our sample size is small and larger models introduce overfitting problems.

With the addition of the attention module, we observed that ResNet18 and ResNet34 were the best classifiers, with both achieving a correct rate of about 91%, with ResNet18 having the highest sensitivity of 91.11% and ResNet34 having the highest specificity of 92.18%. In contrast, the classification results of ResNet50 and ResNet101 were slightly inferior, with accuracy of 87.66% and 85.55%, respectively.

This result also demonstrates that the mechanism allows the model to focus more on the lesion-related regions during classification, thus improving the classification accuracy. In addition, ResNet18 and ResNet34, as lighter models, were able to obtain better performance after adding the attention mechanism, which was in line with our expectation. For our small amount of data, increasing the depth of the network in deep neural networks may lead to overfitting, reducing the generalization performance of the model, and thus may not be beneficial for accurate image classification.

## 5. Conclusion

Previous work has used deep learning to detect kidney disease from fundus images or retinal images (20–22). However, for complex diabetic nephropathy retinopathy, simple deep learning models may not be accurate enough to identify. In this work, we propose an attention mechanism model based on lesion region perception, which can guide the model to better detect lesion regions by giving them higher weights. This allows for non-invasive screening of diabetic patients at risk for DN. Compared with traditional deep learning models, our model can more accurately focus on the lesion region information of retinal OCT images. This allows the model to ignore irrelevant regions to a certain extent, thus improving the classification accuracy. We conducted experiments on a clinical dataset and the experimental results demonstrated the validity of our proposed model. By experimenting with retinal OCT volume data from diabetic patients, we obtained 91.88% accuracy, 91.11% sensitivity and 92.05% specificity. This indicates that our model plays an important role in screening patients with high-risk DN.

However, our work has some limitations. On the one hand, we used only retinal OCT images for DN screening without considering other imaging modalities. This ignores to some extent the pattern-specific information between different imaging modalities. On the other hand, the high cost associated with the collection, storage, and labeling of medical data often presents us with the challenge of data imbalance and limited data volume. This may affect the generalization performance of the model. In the future, we can consider expanding the number of samples and analyzing other medical imaging data jointly with OCT images. To obtain more comprehensive and accurate image information and further improve the accuracy and reliability of the model.

## Data availability statement

The original contributions presented in the study are included in the article/Supplementary material, further inquiries can be directed to the corresponding authors.

## Ethics statement

Written informed consent was obtained from the individual(s) for the publication of any potentially identifiable images or data included in this article.

## Author contributions

YL: Methodology, Writing – original draft. FZ: Formal analysis, Writing – review & editing. XG: Writing – review & editing. TL: Data curation, Writing – review & editing. JD: Writing – review & editing.
