# Supplementary material for: Lesion-aware attention network for diabetic nephropathy diagnosis with optical coherence tomography images
Source: Front Med (Lausanne). 2023 Oct 27;10:1259478. doi: 10.3389/fmed.2023.1259478 (PMC10641799; doi:10.3389/fmed.2023.1259478)
Supplement: Supplementary file 1 [file Data_Sheet_1.docx]

Supplementary Material

**Lesion-Aware Attention Network for Nephrotic Retinopathy Diagnosis with Optical Coherence Tomography Images**

**Yuliang Liu^1,2^,Fenghang Zhang^1,2^,Xizhan Gao^1,2*^,Tingting Liu^3*^,Jiwen Dong^1,2^**

^1^School of Information Science and Engineering,University of Jinan,Jinan,China

^2^Shandong Provincial Key Laboratory of Network‐based Intelligent Computing,Jinan,China

^3^Shandong Eye Institute,Shandong First Medical University & Shandong Academy of Medical Sciences,Jinan,China

*** Correspondence:**

XizhanGao, School of Information Science and Engineering,University of Jinan,Jinan,China. Tingting Liu,Shandong Eye Institute,Shandong First Medical University & Shandong Academy of Medical Sciences,Jinan,China.

[ise_gaoxz@ujn.edu.cn](mailto:ise_gaoxz@ujn.edu.cn); [tingtingliu@vip.sina.com](mailto:tingtingliu@vip.sina.com)

# Supplementary Figures and Tables

## Supplementary Figures


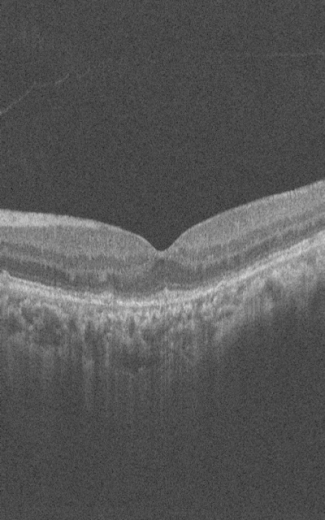

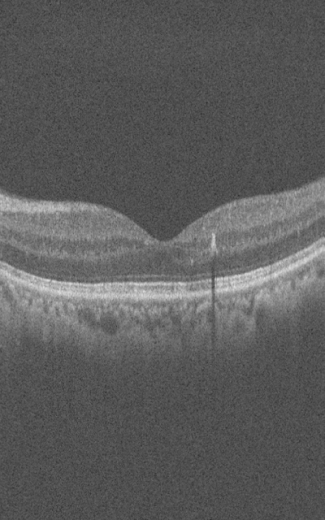

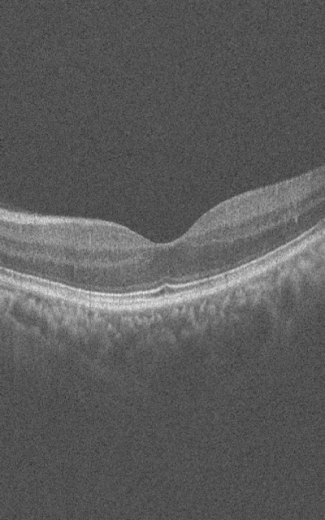


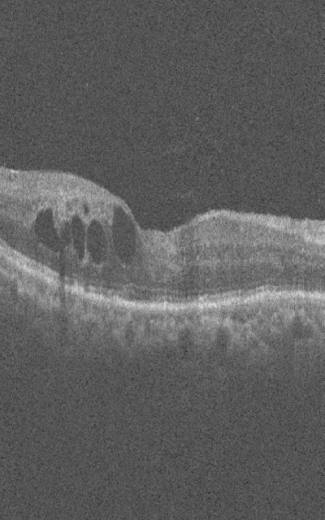

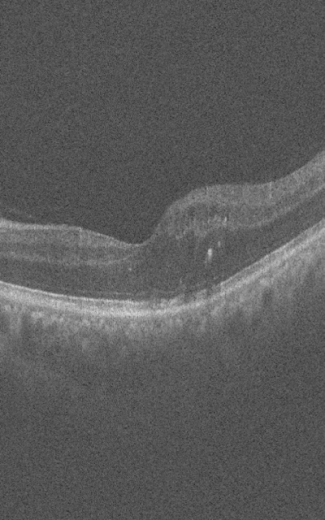

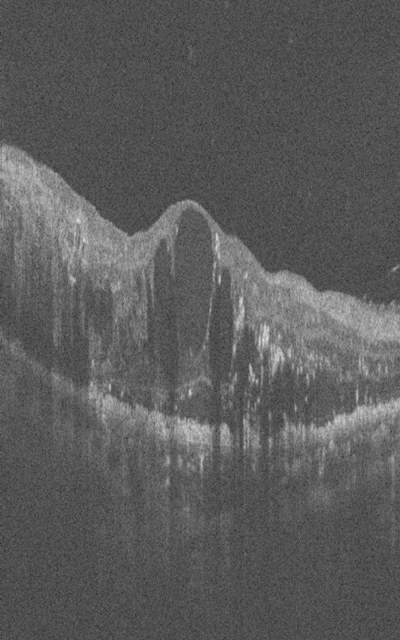


**Figure 1.** The raw dataset can't be freely available in the manuscript, the appendix, nor a publicrepository because of ethical restrictions. We attach 3 pictures of different categories as a supplementary introduction.
